# The Importance of Alexithymia in Post-surgery. Differences on Body Image and Psychological Adjustment in Breast Cancer Patients

**DOI:** 10.3389/fpsyg.2020.604004

**Published:** 2020-12-18

**Authors:** Lorena Gutiérrez Hermoso, Lilian Velasco Furlong, Sofía Sánchez-Román, Lorena Salas Costumero

**Affiliations:** ^1^Department of Psychology, Universidad Rey Juan Carlos, Madrid, Spain; ^2^Department of Neurology and Psychiatry, Instituto Nacional de Ciencias Médicas y Nutrición Salvador Zubirán, Mexico City, Mexico

**Keywords:** breast cancer, body image, coping strategies, psychological adjustment, alexithymia

## Abstract

Breast cancer is a disease that is difficult to face and that often hinders body acceptance. Body changes due to surgery can be very emotionally challenging for those who experience them. The aim of this study is to explore the differences on body image and psychological adjustment on women with breast cancer with high and low alexithymia according to the type of surgery. In this cross-sectional study, 119 women diagnosed with breast cancer (stages I, II, and III) were evaluated with different self-report questionnaires. Afterward, patients were divided into two groups (high and low levels of alexithymia) to analyze dependent variables (body image and psychological adjustment) according to the type of surgery (radical mastectomy or breast conserving therapy). The results of the General Linear Model suggest that when patients show high alexithymia combined with having undergone a radical mastectomy, they show higher levels of Hopelessness. Furthermore, in patients with high alexithymia, higher scores of maladaptive coping styles and greater distortion of body image were found. Alexithymia seems to play an important role in the way in which women cope with their disease, especially in those with radical mastectomy.

## Introduction

The high incidence of breast cancer worldwide has led the medical community to consider this disease as a serious public health problem. Epidemiological data shows more than 2,00,000 new cases in the coming years, and the estimate indicates more than 42,000 deaths due to the disease (American Cancer Society, 2019).

Surgical treatment is one of the options that can be offered today to this group of patients. Radical mastectomy (MSTR) is the removal of the entire breast, generally including the nipple. Although the survival rate can reach up to 40%, MSTR is associated with a high probability of lymphedema in the future, in addition to deformities in the affected chest, arm or shoulder (Turkington and Krag, 2005). Breast conserving therapy (BCT) is a type of surgery performed by removing the tumor and surrounding tissue to prevent its growth, and it is usually followed by adjuvant radiotherapy sessions as a substitute for mastectomy. MSTR surgery is the most commonly used method to remove the affected nodes (Hoover et al., 2011). However, many women’s concerns for body image and femininity lead them to opt for BCT in order to preserve the shape of the affected breast as much as possible (Puts et al., 2015; Gu et al., 2018). BCT surgery has been shown to provide greater psychological benefits in most patients diagnosed with early stage invasive breast cancer (I, II or III), as it impacts their body image differently. Body image is understood as the subjective representation of physical appearance and a source of satisfaction or dissatisfaction when this perception does not match with social ideals (Parker et al., 2007), which is why this construct influences self-esteem (Sarwer and Cash, 2008) and sexuality (Fobair and Spiegel, 2009). The diagnosis of breast cancer is likely to increase the probability of distorting body image. However, surgery is one of the factors responsible for the negative body changes that generally cause failed attempts to preserve the physical image (e.g., breast reconstruction) (Sheehan et al., 2007; Zimmerman et al., 2010; Collins et al., 2011) and anxiety related to the new physical condition (Montebarocci et al., 2007; Collins et al., 2011).

A large part of this research was dedicated to studying the relationship between body image and adaptation to the disease in the two types of surgery. Some studies have found that those who have undergone a BCT show an adaptive coping style and the perception they maintain of their own image is more adjusted (Al-Ghazal et al., 2000). Consequently, the quality of life of patients who undergo conservative surgery remains higher compared to those who undergo MSTR (Hoover et al., 2011; Ursaru et al., 2014). However, the results are still controversial, as there are other studies that support the idea that both MSTR and BCT promote maladaptiveness equally in patients (Parker et al., 2007).

Emotional adaptation is intimately linked with coping strategies (Velasco et al., 2020). The emotional distress experienced after breast cancer surgery can be understood by the difference between the ideal physical state of oneself (e.g., good conditioning and physical attractiveness) and the real image (Helms et al., 2008). Premature death or the sudden changes that may occur in their lives due to cancer are usually the type of negative thoughts that increase an altered self-assessment of body image and lead to dissatisfaction with themselves (Przezdziecki et al., 2013). In this context, low adaptation is defined by manifested anxiety and depression (Gregorowitsch et al., 2018) as a consequence of stress caused by personal insecurity, which explains why strategies focused on cognitive avoidance, helplessness or persistent worries tend to be the most common to deal with the disease. These coping styles increase the incidence of physical symptoms such as pain, fatigue, insomnia or loss of limb functionality (specifically, in the arm or armpit of the affected breast), which in turn, influence the probability of recurrence (Kvillemo and Bränström, 2014; Ahadzadeh and Sharif, 2018).

While there is strong evidence that emotional involvement alters overall coping with breast cancer (Parker et al., 2007), less is known about how some constructs that define more stable personality traits can influence efforts dedicated to alleviating the perceived stress associated with the type of surgery and the distortion of the body image of the patients. It is important to understand that the problems that arise in adapting to the continuous changes that the disease causes usually stem from difficulties at the emotional level (Kojima, 2012). Alexithymia, defined as the inability to recognize and express one’s emotional states and those of others, leads to numbness in affective regulation and causes an externally oriented thought pattern (Taylor and Bagby, 2012). In general population, these emotional difficulties are more frequent in people who frequently experience negative emotions than in those who experience positive emotions (De Berardis et al., 2017). Consequently, the appearance of psychiatric problems such as anxiety and depression are highly probable (Carano et al., 2012; De Berardis et al., 2017; Serafini et al., 2020) and, therefore, increases the risk of suicide (De Berardis et al., 2020). In this context, alexithymia has become a factor of special interest as it is considered a risk factor in those people who have previously attempted suicide (Hintikka et al., 2004; Huen et al., 2015). In an oncology population, the studies carried out by Luminet et al. (2007) and De Vries et al. (2012) have confirmed that alexithymia is stable over time and that it increases in the presence of anxious and depressive states. Furthermore, it seems to be related to the way in which patients with cancer and other diseases describe their experience of pain, as well as to maladaptive coping and unhealthy behaviors such as a sedentary lifestyle (Porcelli et al., 2007; Landstra et al., 2013). A study led by Kim et al. (2013) point to depressive symptoms, anxiety and advanced stage of cancer as the factors that influence suicidal thoughts 1 year after surgery. One of the explanations provided by the authors focuses on the difficulties suffered by patients as a result of the body changes caused by breast surgery. The lack of acceptance toward the disease and the physical rejection favor that suicide is considered as an alternative solution to the problems they are experiencing. In relation to body image, some studies have indicated body dissatisfaction as a predictor of suicidal ideation in other clinical populations (Brausch and Decker, 2014; Perkins and Brausch, 2019). However, more research is needed to explain alexithymia’s influence as a personality factor that damages body image and promotes the use of maladaptive coping strategies as possible risk factors for women with breast cancer (Saita et al., 2015). As shown, different studies have analyzed body image and psychological adjustment in patients with breast cancer. But despite the variables studied in this population, little is known about their relation to distinct types of surgery in patients with breast cancer in early phases (stages I, II or III). Going one step further, by addressing alexithymia as a relevant variable in this disease, the aim of the present study is to explore the differences between body image and psychological adjustment on women with breast cancer with high and low alexithymia according to the surgery. We addressed this objective by comparing two groups of patients depending on the surgery (MSTR vs. BCT) and on the level of alexithymia (high alexithymia vs. low alexithymia). Basing ourselves on current literature, we hypothesized that women undergoing MSTR will present a more distorted body image and use maladaptive coping strategies in function of alexithymia levels compared to women undergoing BCT.

## Materials and Methods

### Participants

A total of one hundred and thirty-nine women entered as study participants, but the response rate was 85.61%. Women that did not completely fill in all the questionnaires were excluded, leading to a final sample of one hundred and nineteen women, aged 18 years and older. Participants were diagnosed with invasive breast cancer in early stages (stage I, II or III) and they underwent surgery (MSTR or BCT). Patients were recruited from the National Institute of Medical Sciences and Nutrition Salvador Zubirán (México) and Hospital Universitario de Móstoles (Spain). This study was approved by the Committee on Ethics of the collaborating institutions and data collection was carried out between October 2015 and July 2019. Then, the researchers contacted patients interested in participating in order to perform an individual evaluation in person. The inclusion criteria established for the study were: (a) being over 18 years old, (b) having a diagnosis of invasive breast cancer in stages I to III, (c) having undergone surgery for the removal or reduction of the malignant tumor, and (d) signing the informed consent form. The exclusion criteria consisted of: (a) presenting a diagnosis of invasive breast cancer in the metastatic phase, (b) presenting a comorbid diagnosis with a mental illness, mental retardation or cognitive disorder, and (c) presenting any disability that prevented them from completing the evaluation questionnaires. This information was confirmed with the medical record.

The average age for participants was 53.16 (*SD* = 10.30). Ninety women were Mexican (75.6%) and twenty-nine were Spanish (24.4%). Sixty-six were married or living with a partner (55.4%), 13 were separated or divorced (10.9%), and 8 were widowed (6.7%). One participant had no educational studies (0.8%), 24 participants had primary studies (20.1%), 37 completed high school or secondary studies (31.1%) and 57 women had university level education (48%). Forty-three women were housewives (36.4%), 49 women were working (41.4%) and 27 women were unemployed (22.2%). No significant differences were found according to nationality and stage of diagnosis regarding the study variables (alexithymia, body image, and coping strategies).

With respect to the clinical variables, participants reported an average time of diagnosis at 21.62 months (*SD* = 3.07). This means that, at the time of the evaluation, there were some patients who were in the process of the disease and others who had been recently diagnosed. On the other hand, there are also women who were in the disease-free phase (being currently treated with hormone therapy for 5 years for relapse prevention). Regarding the stage of disease, forty-eight women were in stage II (40.3%), thirty-nine women in stage I (32.8%) and thirty-two women in stage III (26.9%). Radical mastectomy was the most widely used surgery (*n* = 71, 59.7%) followed by BCT (*n* = 48, 40.3%). Finally, hormonal treatment was the most common form of treatment (*n* = 55, 46.2%), following chemotherapy (*n* = 50, 42%) and radiotherapy (*n* = 14, 11.8%).

### Procedure

Participants were recruited by physicians from the reference Oncology units based on inclusion and exclusion criteria, and the researchers then called to make an appointment at dedicated facilities for individual evaluations. After a brief interview to collect data about medical history, the participants completed the questionnaires described below.

### Measures

#### Sociodemographic and Clinical Data

An “*ad hoc*” questionnaire was used including age, marital status, educational level, and working status as socio-demographic variables, and time since diagnosis, stage of disease, type of surgery and medical treatment as clinical variables.

#### Alexithymia

The Spanish version of the Toronto Alexithymia Scale (TAS-20) was used (Martínez-Sánchez, 1996). It is composed of 20 items with a Likert-type response, which shows the degree of agreement or disagreement with each statement. Scores range between 20 and 100. Equal or higher scores than 61 indicates a high level of emotional difficulty (Páez et al., 1999). In addition to evaluating the level of global alexithymia, it also provides information on three factors within the construct: difficulty in identifying and discriminating emotional cues, difficulty in verbal expression of emotions and feelings, and externally oriented thinking. Its internal reliability is adequate and good for most of the translations (Martínez-Sánchez, 1996) and the stability and replicability of its factor structure has been confirmed in both clinical and non-clinical samples (Bagby et al., 1994; Páez et al., 1999). According to previous research, we used the global score to explore the role of alexithymia as a stable and characteristic construct of personality in the participants (Baudic et al., 2016; Marrazzo et al., 2016). For this scale, Cronbach alpha was 0.84.

#### Body Image

The Hopwood Body Image Scale (Hopwood et al., 2007) was used. This self-report does not have a consolidated validation in the Spanish-speaking population, but previous reviews confirmed that the instrument has good validity for the population with breast cancer (Sebastián et al., 2007). High scores indicate a high distortion in body image. According to Rosenberg (1965), knowing the relationship that one feels with one’s physical appearance is part of the self-concept (set of judgments and evaluations of oneself regarding one’s own behavior, abilities or appearance) and self-esteem, which are the set of positive or negative feelings that we have about ourselves. The instrument has a high internal consistency. In our sample, we found α = 0.89.

#### Psychological Adjustment

To identify the coping strategies, the Spanish version of Mental Adjustment to Cancer Scale (MAC) was administered (Ferrero et al., 1994). Coping is conceptualized as the set of personal resources to face the demands of one’s environment, in this case, the demands related to the disease (Lazarus and Folkman, 1984). The factor structure of the scale includes five coping strategies: fighting spirit, hopelessness/helplessness, anxious preoccupation, fatalism, and avoidance. People who maintain a coping style based on the spirit of struggle as a strategy, perceive the disease as a challenge that benefits them in adopting the experience as an opportunity for personal growth. The coping style centered on the hopelessness/helplessness strategy is defined by high levels of anxiety and depression along with low situational control, which causes a person to make decisions with a lack of regard for their health. Anxious preoccupation is a strategy based on high levels of anxiety and recurring concerns regarding the diagnosis and any demands related to the treatment to follow. People who tend to use a fatalism-based coping strategy often give up when faced with challenging circumstances. Finally, the avoidance-based coping style focuses on the tendency to seek peace of mind without getting too informed about the disease. In some clinical studies with non-Hispanic populations, they have confirmed that the use of maladaptive strategies such as hopelessness and anxious preoccupation are effective indicators of the degree of maladjustment and psychological distress (Grassi et al., 2005). According to the original version of the instrument, high scores indicate greater use of these strategies. It has an internal consistency, with an alpha of Cronbach ranging between 0.65 and 0.78. In the present study, the alpha of Cronbach ranged from 0.70 to 0.78.

### Statistics

Analyses were performed using the SPSS 22 Statistics Package (Armonk, NY, United States). The descriptive analyses of the total sample were calculated according to the sociodemographic and clinical variables. Internal consistency analysis was performed according to Cronbach’s alpha coefficient. Comparisons between means were performed with Student’s *t*-test in both groups (high and low alexithymia and both surgeries). The objective of this study was to determine the effects of the level of alexithymia and the type of surgery on body image and coping strategies, as well as the interaction between the level of alexithymia^∗^ and type of surgery. To do this, we conducted six 2 × 2 factorial design ANOVAs with the between-group factor condition (high or low alexithymia) and type of surgery (MSTR or BCT), with one for each dependent variable (body image, fighting spirit, helplessness, anxious preoccupation, fatalism, and avoidance).

## Results

### Differences Between Alexithymia and Surgery Groups on Dependent Variables

As shown in Table 1, according to alexithymia levels, there are significant differences on body image scores and the following maladaptive coping strategies: helplessness, anxious preoccupation, fatalism, and avoidance. The distortion on body image is greater in the group of women with high alexithymia compared to women with low alexithymia (*t* = −4.08, *gl* = 63.87). Likewise, the use of maladaptive coping strategies is more pronounced in women with high alexithymia compared to women with low alexithymia. Specifically, helplessness is greater in the group of high alexithymia compared to women with low alexithymia (*t* = −5.05, *gl* = 54.95), anxious preoccupation is highest in women with high alexithymia compared to low alexithymia group (*t* = −2.17, *gl* = 100.53). Fatalistic perception is again more accentuated in high alexithymia group compared to the group with low alexithymia (*t* = −2.60, *gl* = 104.73) and, finally, avoidance is higher in women with high alexithymia compared to women with low alexithymia (*t* = −2.86, *gl* = 66.67).

**TABLE 1:** Differences between alexithymia and surgery groups on dependent variables.

	**Alexithymia**			**Surgery**		
	**High alexithymia (≥61) Mean (*SD*) (*n* = 39)**	**Low alexithymia (<61) Mean (*SD*) (*n* = 80)**	***p***	**Radical mastectomy Mean (*SD*) (*n* = 71)**	**Breast conserving therapy Mean (SD) (*n* = 48)**	***p***
Alexithymia				51.47(11.46)	54.36(10.94)	0.050*
Body image	15.07(8.24)	5.05(3.09)	0.000***	11.97(3.70)	9.32(3.58)	0.050*
Fighting spirit	48.09(7.66)	47.73(10.29)	0.845	47.09(10.18)	47.96(8.62)	0.664
Helplessness	12.28(4.89)	8.34(3.44)	0.000***	9.67(2.59)	9.32(3.20)	0.674
Anxious preoccupation	24.70(4.52)	22.15(6.54)	0.016*	22.27(5.97)	23.54(5.32)	0.266
Fatalism	21.17(3.89)	18.39(5.98)	0.003**	19.33(6.02)	18.83(5.48)	0.641
Avoidance	2.36(1.21)	1.72(1.10)	0.007**	1.91(0.19)	1.93(0.15)	0.060

*p ≤ 0.05; **p ≤ 0.01; ***p ≤ 0.001.

Regarding the type of surgery, women who have undergone BCT have higher alexithymia scores compared to women with a MSTR (*t* = −0.938, *gl* = 110.57) and the surgery that most affects body image is MSTR compared to BCT (*t* = 1.88, *gl* = 115.43).

### Interaction Effect on Dependent Variables and the Type of Surgery in Breast Cancer Patients in Function of Alexithymia Levels

Table 2 shows the results of the factorial ANOVA for the dependent variables. As it can be seen, statistically significant differences were found in body image and all coping strategies (helplessness, anxious preoccupation, fatalism, and avoidance) except fighting spirit, in function of alexithymia as an independent factor (group). Also, significant differences were found on body image in relation to surgery and interaction effects were only significant for helplessness.

**TABLE 2:** Factorial ANOVA (2 × 2) for each dependent variable.

	***F***	***p***	**η*^2^***	**Observed power**
**Body image**				
Corrected model	8.57	0.000***	0.184	0.993
Group (low alexithymia <61, high alexithymia ≥61)	14.85	0.000***	0.115	0.969
Type of surgery	7.11	0.009**	0.059	0.754
Alexithymia*Type of surgery	3.49	0.064	0.030	0.457
Adjusted *R*^2^	0.163			
**Fighting spirit**				
Corrected model	0.065	0.978	0.002	0.061
Group (low alexithymia <61, high alexithymia ≥61)	0.009	0.965	0.000	0.051
Type of surgery	0.003	0.960	0.000	0.050
Alexithymia*Type of surgery	0.157	0.692	0.001	0.068
Adjusted *R*^2^	−0.025			
**Hopelessness/Helplessness**				
Corrected model	10.37	0.000***	0.214	0.998
Group (low alexithymia <61, high alexithymia ≥61)	22.03	0.000***	0.162	0.996
Type of surgery	2.08	0.152	0.018	0.298
Alexithymia*Type of surgery	4.37	0.039*	0.037	0.545
Adjusted *R*^2^	0.194			
**Anxious preoccupation**				
Corrected model	1.77	0.155	0.045	0.453
Group (low alexithymia <61, high alexithymia ≥61)	4.31	0.040*	0.036	0.539
Type of surgery	0.480	0.488	0.004	0.106
Alexithymia*Type of surgery	0.032	0.859	0.000	0.054
Adjusted *R*^2^	0.020			
**Fatalism**				
Corrected model	2.60	0.155	0.056	0.064
Group (low alexithymia <61, high alexithymia ≥61)	6.05	0.040*	0.015	0.050
Type of surgery	0.974	0.488	0.326	0.008
Alexithymia*Type of surgery	0.412	0.859	0.522	0.004
Adjusted *R*^2^	0.039			
**Avoidance**				
Corrected model	3.34	0.022*	0.081	0.746
Group (low alexithymia <61, high alexithymia ≥61)	6.69	0.011*	0.055	0.728
Type of surgery	1.83	0.627	0.002	0.077
Alexithymia*Type of surgery	1.83	0.178	0.016	0.269
Adjusted *R*^2^	0.057			

*p ≤ 0.05; **p ≤ 0.01; ***p ≤ 0.001.

**FIGURE 1:**
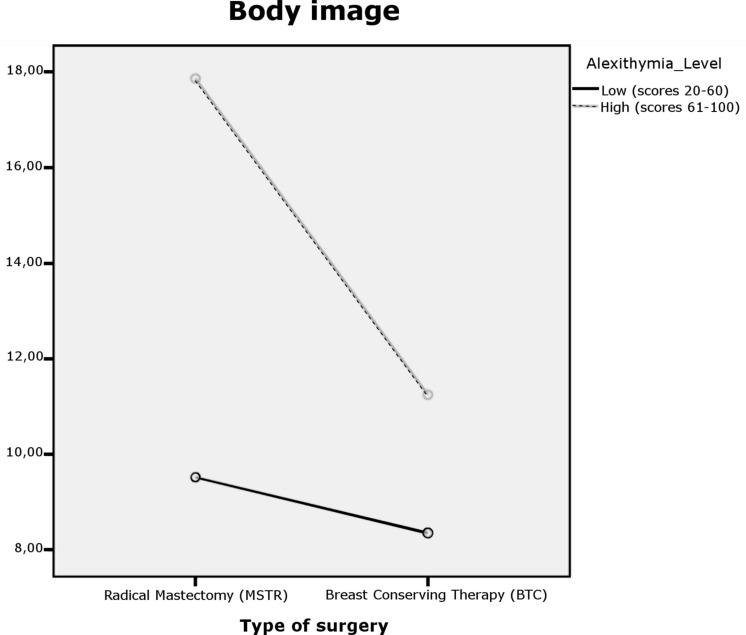
Dual interaction graph for body image.

For body image, significant effects were found for group (*p* = 0.000) and type of surgery (*p* = 0.009), and close for the interaction effect (*p* = 0.064). The model explains 16.3% of the variability in body image. Table 3 shows the simple effects that compare the effects of presenting high or low alexithymia in the two types of surgery. All comparisons were made using the Bonferroni adjustment to control the type I error rate. As shown in Figure 1, women who have undergone a MSTR have higher scores for body image distortion, these scores are considerably higher in those participants who also have high alexithymia compared to women who have low alexithymia.

**TABLE 3:** Simple effects: comparison between high and low alexithymia groups in different type of surgery.

	**Mean difference**	**Std. Error difference**	**Sig.**
**Body image-Type of surgery**			
Radical mastectomy	8.34	1.87	0.000***
Breast conserving therapy	2.89	2.23	0.197
**Helplessness-Type of surgery**			
Radical mastectomy	5.31	1.00	0.000***
Breast conserving therapy	2.03	1.19	0.092

***p ≤ 0.001.

**FIGURE 2:**
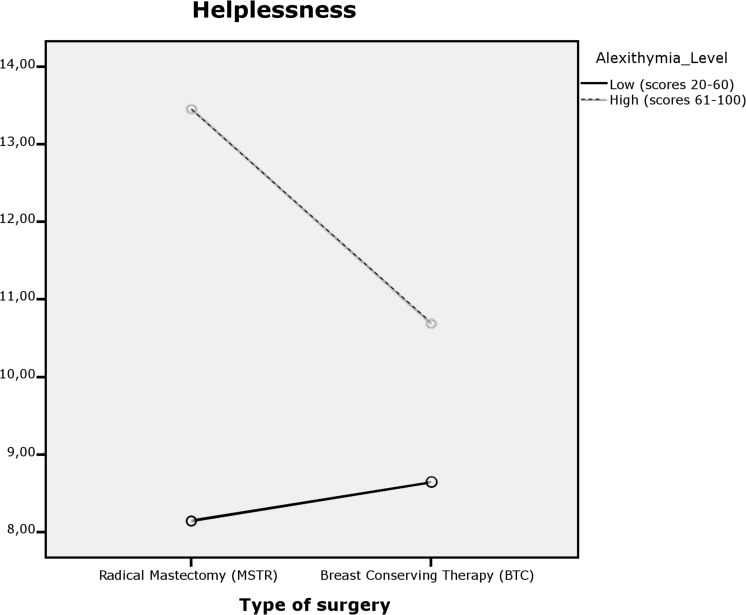
Dual interaction graph for helplessness.

For hopelessness/helplessness, the main effects were found for the group (*p* = 0.000) as well as the interaction effect (*p* = 0.039) but not for type of surgery (*p* = 0.152). The model explains 19.4% of the variability of helplessness. Figure 2 illustrates the results obtained. Women who have undergone a MSTR have higher scores in this coping strategy, and these scores were even higher in those participants who have high alexithymia. In addition, as shown in Table 3, the type of surgery factor indicates significant differences in helplessness for the group that has undergone MSTR (*M* = 9.67, *SD* = 2.59). These differences were analyzed employing the Bonferroni index.

For anxious preoccupation, only significant effects are observed for group (*p* = 0.040), not for type of surgery (*p* = 0.488) or the interaction effect (*p* = 0.859) (see Table 2). Thus, the significant main effect of group factor indicates differences in anxious preoccupation between women with high alexithymia (*M* = 24.70, *SD* = 4.52) and with low alexithymia (*M* = 22.15, *SD* = 6.54) (*t* = −2.46, *gl* = 100.53). When simple effects are observed, depending on the type of surgery, there are no differences in the high and low alexithymia groups.

With respect to fatalism, the differences found can be observed for group (*p* = 0.040) but not for type of surgery (*p* = 0.488) or interaction (*p* = 0.859) (see Table 2). The main effect of the group indicates that women with low alexithymia (*M* = 18.39, *SD* = 5.987) score lower in fatalism that women with high alexithymia (*M* = 21.17, *SD* = 3.89) (*t* = −3.03, *gl* = 104.73).

Last, for avoidance, only significant effects are observed for group (*p* = 0.011) but not for type of surgery (*p* = 0.627) or interaction effect (*p* = 0.178) (see Table 2). This shows that women with high alexithymia (*M* = 2.36, *SD* = 1.21) obtain higher avoidance scores than women with low alexithymia (*M* = 1.72, *SD* = 1.10) (*t* = −2.86, *gl* = 66.67).

## Discussion

The main objective of this study was to explore the differences on body image and psychological adjustment on women with breast cancer with high and low alexithymia according to the surgery.

In relation to the results obtained, interaction effects were found only for the Hopelessness/Helplessness strategy. Specifically, high alexithymia and having undergone a MSTR were associated with higher levels of hopelessness. As noted above, hopelessness is a feeling that combines anxiety to achieve unattainable goals at the moment and depression due to the lack of resources to meet those goals (in this case, overcome the disease). In studies carried out with populations with chronic pain, it is observed that alexithymia is related to greater symptoms of anxiety and depression due to the reduced ability to regulate or successfully reduce negative emotions (Aaron et al., 2020). In line with these data, the interaction found between MSTR and alexithymia indicates that women undergoing mastectomies manifest greater difficulties in knowledge about their own emotions. Repression and difficulty in expressing emotions favor the use of strategies based on high levels of anxiety and depression, such as the style of coping centered on hopelessness/helplessness.

The study by Andreu et al. (2012) suggests that the suppression of emotions related to the adverse changes caused by surgery are usually a recurring short-term control strategy. It has been shown that women who have had mastectomies reported higher hopelessness scores compared to women who received BCT (Den Oudsten et al., 2009). Our results support the conclusions described by these authors and, furthermore, emphasize that the role of alexithymia is important in understanding maladjustment in coping that women with breast cancer who have undergone one type of surgery or another experience.

When analyzing coping styles, it was found that hopelessness, anxious preoccupation, fatalism and avoidance had significantly higher scores in women with high alexithymia when compared with those with low alexithymia. No significant differences were found in any of the coping styles between the two types of surgery. According to previous studies, women with breast cancer constantly remember situations that make them suffer and reflect on the negative emotions that these situations awaken in them (Baider and De−Noor, 1997). According to the transactional model of stress developed by Lazarus and Folkman (1984), the disease is perceived as a source of threat that causes negative emotional states. The inability to identify and express these emotional states can leave patients unable to effectively reduce anxiety, such as anxious preoccupation (based on high levels of anxiety), hopelessness/helplessness strategy (based on high levels of anxiety) and depression, fatalism (based on lack of acceptance of circumstances) and avoidance (based on low levels of anxiety and depression). Therefore, the coping pattern of breast cancer patients who present high alexithymia is usually considered maladaptive, since it increases the perception of threat and blocks the implementation of personal resources. In addition, it can increase suicidal ideation as indicated by previous literature on the relationship of negative emotional state with suicidal thoughts in different clinical populations (Carano et al., 2012; De Berardis et al., 2017; De Berardis et al., 2020; Serafini et al., 2020).

Regarding body image, the alexithymia-type of surgery interaction was not found to have an effect on it, although it did independently associate them both. In this sense, it is observed that the surgery affects the perception that each of the participants has about their body, being more distorted in those women who have undergone a mastectomy. However, the ignorance in the identification of the emotional state does not affect the fact that this group of participants who have undergone one or another type of surgery perceives their body image more distorted.

Studies explaining body image distortion in breast cancer patients highlight the role of surgery as a relevant variable (Moyer, 1997; Al-Ghazal et al., 2000; Jabłoński et al., 2018), since the area of the breasts is especially threatened. It is important to highlight that the chest is a part of the body closely linked to sexuality, femininity and the identity attributed to the sense of being a woman (Carver et al., 1998; Petronis et al., 2003; Moreira and Canavarro, 2010; Jabłoński et al., 2018). Body image distortion involves feeling ashamed of body changes (e.g., breast scar, weight gain, loss of function or loss of sensitivity), leading to rejection and problems in sexual intercourse (Han et al., 2010; Jabłoński et al., 2018). Other studies go a step further and specify which type of breast operation causes psychological sequelae (Moyer, 1997; Janni et al., 2001; Parker et al., 2007). Similar to our results, the authors of the previous studies have identified that, in general, patients with breast cancer tend to perceive the body distorted after surgery, the affection of body image in women undergoing an MSTR being more pronounced compared to women who have had a BCT. In line with these results, the findings of the present study confirm that high levels of alexithymia increase thoughts related to rejection toward the body in both groups. Despite the limited empirical background on the impact of alexithymia on body image, the significant effect between these two variables also supports the results of studies carried out with other clinical and non-clinical populations on the influence of alexithymia on body control. In these studies conducted with healthy populations, it is suggested that the difficulty in distinguishing emotional states reinforces the focus of attention on negative aspects of the body, which exacerbates the alterations in body image (Montebarocci et al., 2006; De Berardis et al., 2007). The results found in the participating sample support the conclusions obtained in the previous studies with other clinical populations from the general linear model.

This study has a series of limitations derived from, above all, the type of study. As it is a cross-sectional design, this prevents us from obtaining stable conclusions over time. It would be necessary to develop longitudinal studies to confirm the maintenance of these results over time. Additionally, in the collected data it’s not known if the study participants have already undergone or will undergo breast reconstruction, and it would be important to consider this data, since the psychological impact on body image and coping strategies may vary. According to the previous literature, emotional difficulty is a psychological characteristic that defines cancer population. However, there are few studies that make a comparison between healthy population and cancer population. In this study we used only the global scale to explore the role of alexithymia, future research should also explore the role of the subscales (difficulty in identifying emotions, difficulty in emotional expression, and externally oriented thinking) to identify which of them may also be influencing the distortion of body image and maladaptive coping. In relation to this study, it would be necessary to perform a similar statistical procedure that includes a healthy control group. In this way, it will be possible to confirm if the use of maladaptive strategies is common in breast cancer patients.

There are also several strengths in the present study. First, the comparison of the two types of surgery most commonly performed in early-stage breast cancer and which, as has been proven in the results, also suffer sequelae to a psychological level that prevents them from adaptively facing the disease. Alexithymia has been included in the study variables as a construct that has received little attention from clinical research, and which has been shown to interact with the type of surgical intervention. Expanding knowledge about the coping styles of women with breast cancer allows detection of threatening perceptions of the disease. Finally, the study participants have two different nationalities (Spain and Mexico), allowing the study to conclude that the difference in countries does not interfere with the psychological variables of interest. This benefits the generalization of data in Spanish-speaking populations in future studies replicating this methodology with the intention of improving the limitations found.

In conclusion, the results revealed that alexithymia, as a characteristic personality factor of the sample of participants, plays an essential role in the distortion of body image and in coping with the disease. Lack of knowledge in regulation and emotional identification has consequently the use of maladaptive coping styles. The interaction found with the type of surgery performed together with alexithymia have facilitated an in-depth knowledge of the effect of body image and helplessness and hopelessness as the most prevalent feelings in the participants. To our knowledge, there are no other studies that analyze the interaction of clinical variables such as surgery with personality variables such as alexithymia. For this reason, this study offers findings in clinical practice emphasizing the importance of attending to the emotional needs of patients, empowering by reinforcing the use of existing personal resources and incorporating new resources that benefit them in perceiving the disease as a positive challenge.

## Data Availability Statement

The original contributions presented in the study are included in the article/supplementary materials, further inquiries can be directed to the corresponding author.

## Ethics Statement

The studies involving human participants were reviewed and approved by the Universidad Rey Juan Carlos and the Instituto Nacional de Ciencias Médicas y Nutrición Salvador Zubirán. The patients/participants provided their written informed consent to participate in this study.

## Author Contributions

LG, LV, and SS-R conducted the evaluation of methodology, supervisions, drafting the manuscript, and revisions for important intellectual content. LG and LS performed data collection, analysis and administrative, technical, and material support. All authors of this study generated the initial research questions, developed the research protocol and defined the inclusion and exclusion criteria.

## Conflict of Interest

The authors declare that the research was conducted in the absence of any commercial or financial relationships that could be construed as a potential conflict of interest.

## Abbreviations

BCT: breast conserving therapy
MSTR: radical mastectomy.
